# Role of Tele-ultrasound for Teaching Ultrasound-guided Nerve Blocks in the Emergency Department: A Case Series from Peru

**DOI:** 10.5811/cpcem.2022.2.55417

**Published:** 2022-07-27

**Authors:** David A. Martin, Marco Guillen, Angel Farro, Maribel Condori, Andrea Dreyfuss, Arun Nagdev

**Affiliations:** * Highland Hospital - Alameda Health System, Department of Emergency Medicine, Oakland, California; † EsSalud Cusco: Hospital Nacional Adolfo Guevara Velasco, Department of Emergency Medicine, Cusco, Peru; ‡ Hospital Nacional Dos de Mayo, Parque “Historia de la Medicina Peruana.” Department of Emergency Medicine, Lima, Peru; § Hennepin County Medical Center, Department of Emergency Medicine, Minneapolis, Minnesota

**Keywords:** *tele-ultrasound*, *point-of-care ultrasound*, *emergency department*

## Abstract

**Introduction:**

Ultrasound-guided nerve blocks (UGNB) represent a procedural skill set that can be used to treat acute pain by physicians in the emergency department (ED). However, limited access to education and training represents a barrier to widespread adoption of this core skill set. The implementation of UGNBs within the ED can aid in resource allocation, particularly in limited-resource settings.

**Case Series:**

In this case series we discuss our experience using tele-ultrasound to train emergency physicians on the use of UGNBs within our international point-of-care ultrasound fellowship in Peru. We highlight the potential role UGNBs serve in management of acute pain when working in resource-limited, public safety-net hospitals in Peru.

**Conclusion:**

Tele-ultrasound may represent a strategy for teaching procedures such as UGNBs via remote guidance and supervision.

## INTRODUCTION

Ultrasound-guided nerve blocks (UGNB) are a critical part of the multimodal armamentarium for emergency physicians (EP) when treating acute, painful injuries. The UGNB can reduce reliance on systemic opioids, thereby limiting their potential deleterious side effects.^1^ Additionally, in the emergency department (ED), UGNBs can be used as an alternative to resource- and time-intensive procedural sedation.^2^ The American College of Emergency Physicians recently endorsed a policy statement that states UGNBs represent a core component of multimodal pain management for patients in the ED and are within the scope of practice of EPs.^3^ Despite these advantages, UGNBs have not been widely used in limited-resource settings, and within the United States there is substantial variation among institutions in their use.^4^ We believe that lack of education and training in UGNBs is one of the primary barriers to widespread adoption globally.

Advances in hardware have led to the development of less expensive, handheld ultrasound systems, allowing for greater access to bedside imaging globally. The development of tele-ultrasound on these same systems has allowed for education to be delivered remotely. Moreover, tele-ultrasound has already been shown to be an effective tool for training learners in image acquisition and improving diagnostic accuracy,^5^–^7^ as well as providing real-time procedural guidance.^8^ Collectively, these advances allow for the rapid expansion of bedside imaging and a method to increase remote education.

In our international point-of-care ultrasound (POCUS) fellowship training program in Peru, tele-ultrasound has been an integral part of our educational model.^9^ This was clearly evident during the ongoing coronavirus disease 2019 (COVID-19) pandemic, where remote supervision with tele-ultrasound has been vital in continued education and supervision. With the aid of tele-ultrasound over the past three years, we have been able to train numerous clinicians how to safely and effectively perform UGNBs for acute pain management. We herein present our experience in implementing tele-ultrasound education for UGNBs in multiple limited-resource, safety-net hospitals in Peru. We believe that with the growth of the handheld ultrasound market, tele-ultrasound can be an ideal tool to teach procedures such as UGNBs.

## CASE SERIES

### Case 1

**Image 1:**
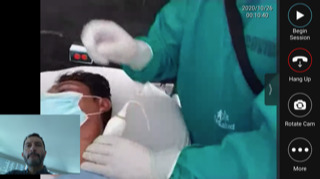
Remote instructor visualizing the ultrasound probe positioning on the patient’s neck.

A 34-year-old man presented with acute onset of left elbow pain after falling from a five-foot ladder. On exam the left elbow was swollen and neurovascularly intact. Plain film radiography demonstrated a closed, displaced left supracondylar fracture. The orthopedic surgeon recommended the patient be placed in a posterior long arm splint and undergo outpatient operative repair; however, oral and intramuscular medications were unsuccessful at reducing the patient’s pain to a tolerable level for splint application. The EP, one of our POCUS fellows in Cusco, Peru, suspected a UGNB could be used to provide analgesia for his patient’s orthopedic injury, so he contacted the on-call group of POCUS fellowship instructors to request assistance with selecting and performing an UGNB. The EP was able to connect via tele-ultrasound with one of the instructors located in the US who recommended performing an ultrasound-guided supraclavicular brachial plexus block. The instructor was able to visualize and guide the ultrasound probe positioning, as shown in Image 1.

**CPC-EM Capsule**

What do we already know about this clinical entity?

*Ultrasound-guided nerve blocks (UGNB) are useful when treating acute injuries in the Emergency Department, but limited access to training inhibits widespread adoption*.

What makes this presentation of disease reportable?

*Data is limited on the use of tele-ultrasound to provide remote, real-time procedural guidance, particularly in the context of global health training programs*.

What is the major learning point?

*Tele-ultrasound can help provide ongoing remote supervision and training for teaching procedures in settings where access to on-site expert support is limited*.

How might this improve emergency medicine practice?

*Emergency physicians can use UGNBs in low-resource settings to improve pain control, and tele-ultrasound can help scale up access to training*.

The EP was able to identify the supraclavicular brachial plexus and successfully inject 20 milliliters (mL) of 0.5% bupivacaine using real-time needle visualization by the remote instructor. The patient’s pain significantly improved at 20 minutes, and he was able to tolerate splint application. He was subsequently discharged home on an oral pain regimen.

### Case 2

**Image 2:**
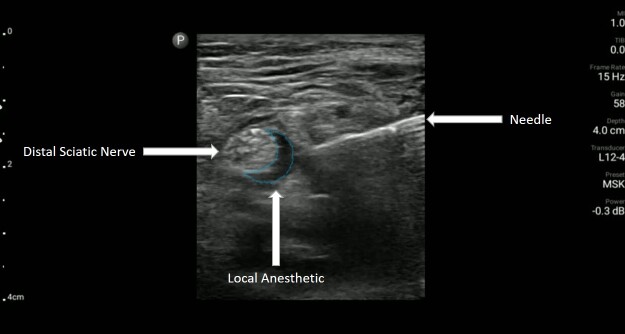
Ultrasound image showing the distal sciatic nerve with surrounding local anesthestic and needle visualized using an in-plane approach.

A 57-year-old man with poorly controlled diabetes presented to the ED with severe foot pain due to a worsening right-sided diabetic heel ulcer over the prior 10 days. On exam, there was concern for bacterial superinfection and the podiatry service was consulted, which recommended operative debridement. Unfortunately, there was a severe backlog of surgical cases, which meant the patient would likely have to wait several days for operative debridement. The EP consulted one of the ultrasound fellowship directors who proposed performing a distal sciatic UGNB based on the location of the diabetic foot ulcer. The EP was able to perform an UGNB she had never previously performed with the help of the remote guidance provided via the tele-ultrasound software on the handheld device. She was able to identify the distal sciatic nerve and injected 20 mL of bupivacaine 0.5% after hydro-dissecting open the potential space with 0.9% normal saline as seen in Image 2.

The patient underwent surgical debridement of the diabetic heel ulcer in the ED and was subsequently admitted to the medicine service for treatment with intravenous antibiotics.

### Case 3

**Image 3:**
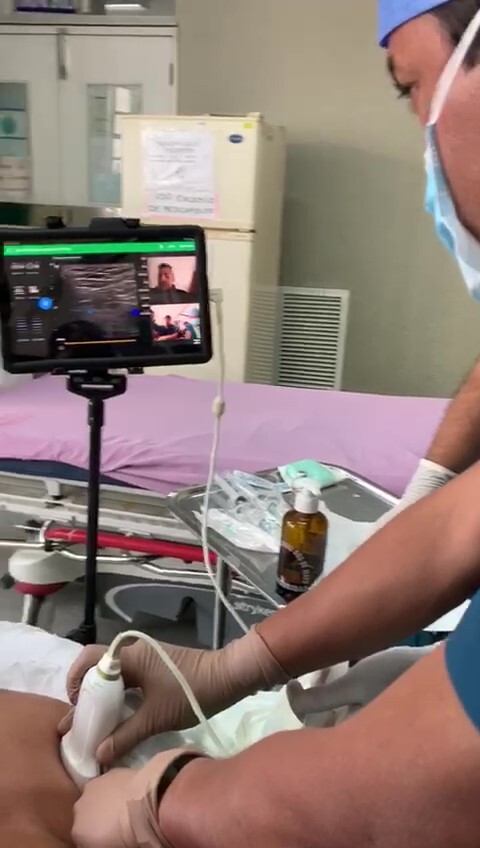
Ultrasound positioning while tele-ultrasound is being employed to provide live needle guidance.

A 35-year-old-man was brought to the ED by ambulance after being struck by a vehicle at low speed, complaining of acute onset of right upper arm pain. On exam, the patient had an obvious deformity of the right upper arm that was neurovascularly intact. Plain film radiography revealed a right-sided displaced, mid-shaft humeral fracture. The patient was placed in a coaptation splint but continued to endorse severe pain despite receiving oral and intramuscular pain medication. The EP consulted with the on-call instructor who recommended a retroclavicular brachial plexus block. Although the EP had never previously performed this UGNB, through the use of tele-ultrasound software, an instructor was able to guide the physician to deposit 20 mL of bupivacaine 0.5% in the appropriate space as seen in Image 3.

After the UGNB the patient’s pain reduced from 8/10 to 1/10 severity, and he was subsequently discharged home on an oral pain regimen.

## DISCUSSION

Despite the recognized benefits of UGNBs in the treatment of acute pain within the ED, this mode of analgesia remains underused in low-resource settings.^10^ Ultrasound-guided nerve blocks represent a core procedural skill set for EPs in the US;^3^ however, limited access to education resources represents a challenge both domestically and globally. Tele-ultrasound can be an ideal technological solution for training EPs in the use of UGNBs in the US and globally.

In our POCUS fellowship training program in Peru, we have integrated the use of tele-ultrasound to provide ongoing remote diagnostic and procedural guidance for our trainees on a weekly scheduled basis. We have been able to circumvent geographical barriers and, more recently, travel restrictions due to the COVID-19 pandemic by employing the use of tele-ultrasound to augment hands-on training and provide procedural guidance as demonstrated by this case series.

We acknowledge that the reproducibility of this intervention is contingent on the existence of a baseline knowledge of POCUS applications. Additionally, the use of tele-ultrasound is dependent on the existence of reliable internet connectivity, although we have been able to reliably use our tele-ultrasound software with one of our fellows who was working in a remote city in the Amazon rainforest. Our success in setting up UGNBs within Peru forms part of a larger educational intervention leveraging technological advances to facilitate cross-continental learning. We are currently working on a descriptive study detailing the total number of UGNBs performed since the initiation of our educational intervention. We are hopeful that these cases will highlight the potential impact that tele-ultrasound can have specifically when it comes to providing remote procedural guidance.

## CONCLUSION

Treating acute pain in the emergency department can be challenging, particularly when working in a low-resource setting. Ultrasound-guided nerve blocks can be used by emergency physicians in low-resource settings to provide improved pain control and resource allocation, particularly in the context of ED crowding resulting from the COVID-19 pandemic. Tele-ultrasound can help provide ongoing remote supervision and training for teaching procedures such as UGNBs in settings where there is limited access to on-site expert support.
